# Population knowledge, attitudes and practices towards malaria prevention in the locality of Makenene, Centre-Cameroon

**DOI:** 10.1186/s12936-022-04253-z

**Published:** 2022-08-05

**Authors:** Joel Djoufounna, Roland Bamou, Marie Paul Audrey Mayi, Nelly Armanda Kala-Chouakeu, Raymond Tabue, Parfait Awono-Ambene, Dorothy Achu-Fosah, Christophe Antonio-Nkondjio, Timoléon Tchuinkam

**Affiliations:** 1 Vector Borne Diseases Laboratory of the Research Unit of Biology and Applied Ecology (VBID-RUBAE), Department of Animal Biology, Faculty of Science of the University of Dschang, Dschang, Cameroon; 2 Organisation de Coordination Pour La Lutte Contre Les Endémies en Afrique Centrale (OCEAC), Yaoundé, Cameroon; 3 Ministry of Public Health, National Malaria Control Programme, Yaoundé, Cameroon; 4 Aix Marseille Université, IRD, SSA, AP-HM, UMR Vecteurs-Infections Tropicales Et Méditerranéennes (VITROME), Marseille, France

**Keywords:** Knowledge, Attitudes, Practices, Malaria, Households, Makenene

## Abstract

**Background:**

To contribute to the mission of the National Malaria Control Programme (NMCP) and guide future interventions in Cameroon in general, and in Makenene in particular, this study assessed the knowledge, attitudes and practices of the population of Makenene towards the fight against malaria.

**Methods:**

Using a semi-structured questionnaire, a descriptive cross-sectional household community survey was carried out in randomly selected households in Makenene, a locality situated between forest and savannah ecotypes.

**Results:**

Out of the 413 households surveyed, all (100%) claimed to have heard of malaria with over 94% (n = 391) associating disease transmission with mosquito bites. The main mosquito control tools used in the area were mosquito nets (92.25%). The majority of participants had good knowledge (55.93%; n = 231), good practices (71.67%, n = 296) but moderate attitudes (47.94%; n = 198) towards malaria control and fight. Good knowledge and practices were recorded mostly in educated persons including public servants and students. Good attitudes were adopted mostly by public servants and students of secondary and higher levels of education.

**Conclusion:**

In Makenene, the population exhibits good knowledge and practices towards malaria and its control. However, despite high LLINs ownership and use, people still complain about malaria in the area. Control tools should be monitored, repaired or replaced when necessary to support the achievement of the NMCP mission.

**Supplementary Information:**

The online version contains supplementary material available at 10.1186/s12936-022-04253-z.

## Background

Malaria still remains a real public health problem in the world especially in Africa. In 2020, 241 million cases and 627,000 death were reported worldwide [1]. In Cameroon, malaria affects the whole country with different levels of endemicity according to ecological settings, ranging from hypo-endemic to hyper-endemic areas [2]. With more than 11 million cases per year, malaria is one of the main reasons for consultation in many health centres in Cameroon, especially for children under 5 years and pregnant women [3]. Vector control operations through the use of long-lasting insecticidal nets (LLINs), and rapid patient care based on an early diagnosis and administration of effective artemisinin-based combinations drugs, are among measures employed by the Government to limit the progress of the disease [3].

However, several factors contribute to the underperformance of the current control methods leading to malaria persistence. These include the rapid expansion of vector resistance to insecticides, changes in biting and resting behaviour and the bad utilization of preventive methods [4–7]. Added to this, the social consequences linked to the COVID 19 pandemic are being noted [8, 9]. In fact, during this period, efforts in the health services mostly oriented towards the COVID 19, retort activities in detriments to malaria prevention and treatment. In addition to disruptions in malaria diagnosis and treatment, the fear to catch COVID 19 by population in hospital reduced significantly their visit in case of suspected malaria cases or other diseases during COVID 19 pandemic [10–13], increasing auto medication [14]. Furthermore, the lack of knowledge about the disease is sometimes responsible for an approximate treatment which might lead to other types of resistance such as the emergence of anti-malarial drugs resistance, and results in repeated cases of malaria within the same household [15, 16].

In order to better understand the limits of LLINs deployed so far by public authorities, factors related to human behaviour must be taken into account for an effective behaviour change towards the elimination of the disease [17]. Previous studies in some localities have shown that, people's adherence to malaria control measures depends in part on their intellectual and cultural background [18, 19]. However, this background differs from one locality to another [5, 17–22]. In fact, the level of malaria endemicity in a locality (entomological inoculation rate and malaria infection rate) depends upon environmental factors. This latter vary from a locality to another, and characterize the different facies, which are widely spreaded in Cameroon. The country therefore displays from Southern part to the Northern zone: the rainy equatorial forest with hyperendemic malaria [23, 24], the savannah zone with hyperendemic malaria and the sahelian zone in the North with hypoendemic malaria [2]. Within each of these zones, some microfacies can be defined, where malaria endemicity is modified either by the nature or the human activities such as: highland areas [25, 26], damps and other industrial infrastructures [27], urbanizations, and forest-savannah intermediate localities which is the case of Makenene. The locality of Makenene is prone to imported malaria, because it is a crossroads for travellers from different parts of the country. Moreover, the locality has not benefited from a third mass distribution campaign of LLINs planned for 2019 due to the fact that, the Centre region to which Makenene belongs was devoted to the government for LLINs coverage. Unfortunately, government is been facing financial crisis which delayed the activity contrary to the other regions attributed to the Global Fund where this third campaign was effectively conducted.

Little is known about the transmission of malaria in Makenene, a locality located in a forest-savannah limit in Cameroon, and which has been hosting internal displaced people (IDPs) due to the socio-political crisis in the country for four years now. Based on previous studies carried out in the central region of Cameroon in general and in Yaoundé in particular, transmission in Makenene could be holo-endemic and seasonal with the main vector *Anopheles gambiae *sensu lato [28] and an annual prevalence around 35% [29]. This study was designed to assess the knowledge, attitudes and practices (KAP) of populations towards the fight against malaria in Makenene in order to shed light into aspects relevant to malaria control programmes (LLINs, free treatment of children under 5 years with artemisinin-based combination therapy, intermittent preventive treatment of pregnant women with quarterly single dose of sulfadoxine-pyrimethamine [25]) and contribute to reduce the failure of the control measures.

## Methods

### Study sites

**Fig. 1:**
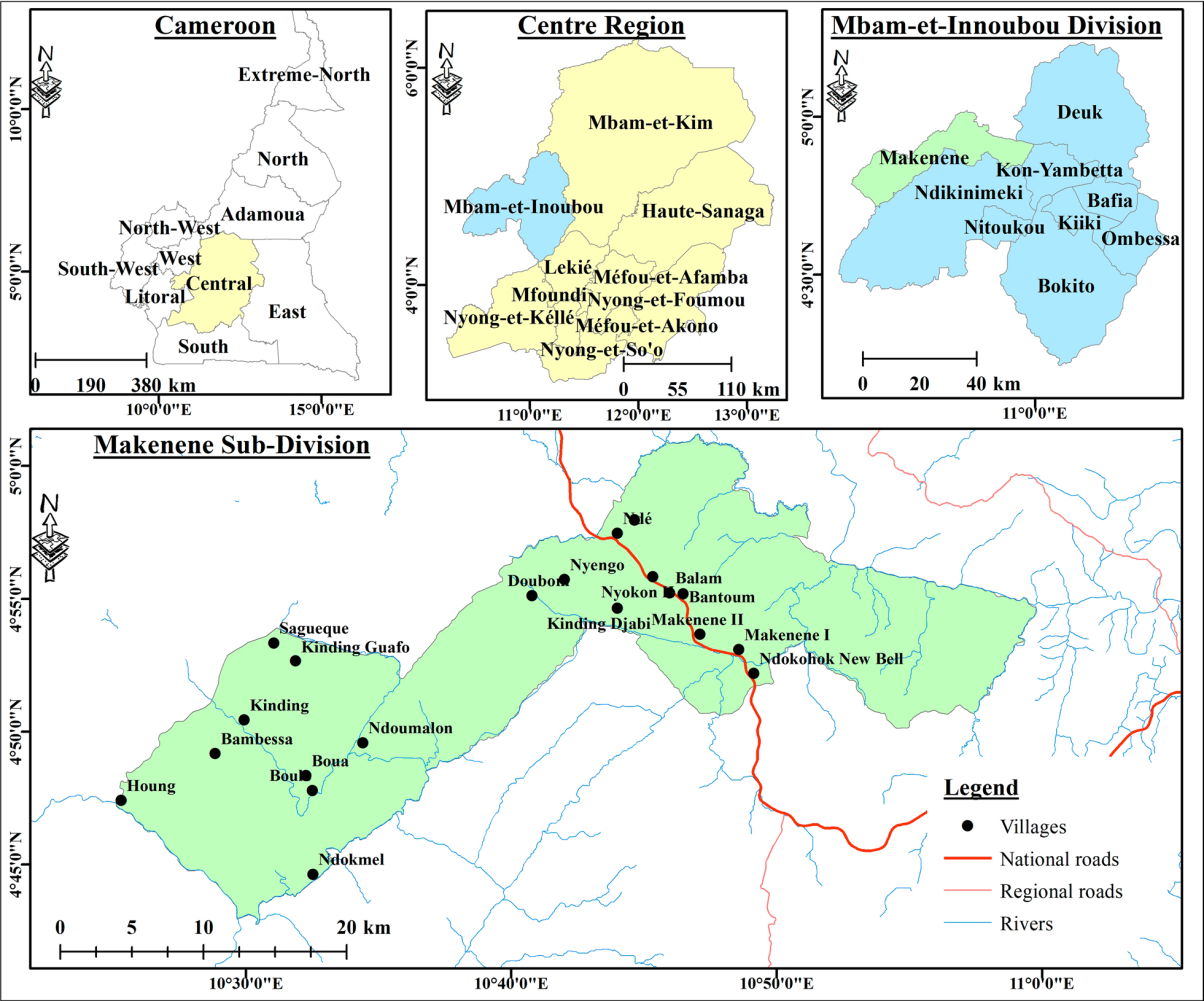
Map of the study site

The study was conducted in the locality of Makenene, Mbam and Inoubou Division, Centre Region of Cameroon (Fig. 1). Located at 580 m of altitude, the locality of Makenene covers an area of 885 km^2^ and is distant from Bafia, the capital of the Division by about 100 km. It is bordered to the north by the Noun River, to the south by the coastal region forest (between 3° 20′ N and 6° N) and to the west and east by the rivers Makenene and Nde (between 9° 40′ E and 13° E) [30]. The Climate in Makenene is equatorial humid and characterized by two dry seasons and two rainy seasons of unequal length (with rainfall up to 721 mm/year): a long dry season (mid-October to March), a small dry season (June to mid-August), a long rainy season (from mid-August to October), and a short rainy season (from March to June) [31]. The population of Makenene is estimated at 35,000 people. The main activity is agriculture to which are added small business, animal husbandry and urban transport by motorbikes among others. The city is full of some basic infrastructures, including health centres (the District Medical Centre of Makenene, the Catholic health centre, the Baptist Church health centre and the Nyokon Integrated health care) schools and public services [32]. The population of Makenene has not benefited from the 3rd mass distribution campaign of mosquito nets scheduled and effective in other regions/localities of the country since 2019.

### Study design and data collection

This was a cross-sectional and descriptive study carried out between 28th July and 20th September 2021. In the absence of the total number of households which unfortunately is lacking in the last census statistical document [33] and no data on the prevalence of malaria in the locality and its surroundings, the sample size was calculated using the Yamane formula [34] as follows: ɲ = N/(1 + N(ε)2); where ɲ is the size of sampling sought; N the population size, and ε the sampling error (5%). According to the last census of DIPAMAK [32], the locality has 35,000 inhabitants. The minimum sample size for this study was estimated at 395 participants but 413 HoH participated to this study.

After obtaining administrative authorizations and ethical clearance, pre-tested questionnaires (a well-designed questionnaire was first randomly pretested on 30 volunteers) were submitted to the HoHs (from randomly selected houses) after obtaining their free and informed consents. In the absence of the HoH, their spouses or adult children (aged above 18 years old) answered the questionnaires. The questionnaire (see Additional file 1), adapted from a copy used for a community survey in Cameroon [5, 19] consisted of a set of 45 questions grouped into 4 levels: (i) socio-demographic characteristics of the studied population; (ii) knowledge of the population about malaria and their mode of transmission; (iii) control/preventive methods applied by the population and, (iv) nocturnal activities of the studied population. Other variables such as the presence or absence of grass and mosquito’s breeding sites around houses and the number of malaria episodes were also recorded.

Household surveys were carried out by a team of trained students and researchers from VBID-URBEA with experience in surveys assisted by trained local community health workers (CHWs). CHWs facilitated the integration in households and helped in the translation of the questionnaire into the local language during the interview/questionnaire survey to people who did not speak neither French nor English.

### Data analysis

After questionnaires administration, responses collected were processed in Microsoft Office Excel version 2016 (see Additional file 2), and analysed using R software version 4.0.4 (R version 4.0.4, 2021-02-15). Frequencies of the different categories of variables were calculated for descriptive statistics and compared using the Chi-square test. Logistic Regression was used to assess the effect of predictive variables (level of education, gender, religion and occupation of the respondent) on good knowledge, good attitudes and good practices (independent variables). Results were considered significant when p < 0.05.

To assess respondents' level of knowledge about malaria, responses to questions on how malaria is transmitted to humans and symptoms of the disease were considered. Participants who answered those two questions correctly (knowledge of the vector and at least two symptoms of the disease) were considered to have good knowledge while those who did not answer those two questions correctly were considered to have poor/bad knowledge. Participants with good attitudes were those who had knowledge about the disease and went to the hospital when they suspected it, whereas those who possessed LLINs, had LLNIs on all beds and used them regularly were considered to have good practices.

## Results

### Sociodemographic characteristics of the study participants

**Fig. 2:**
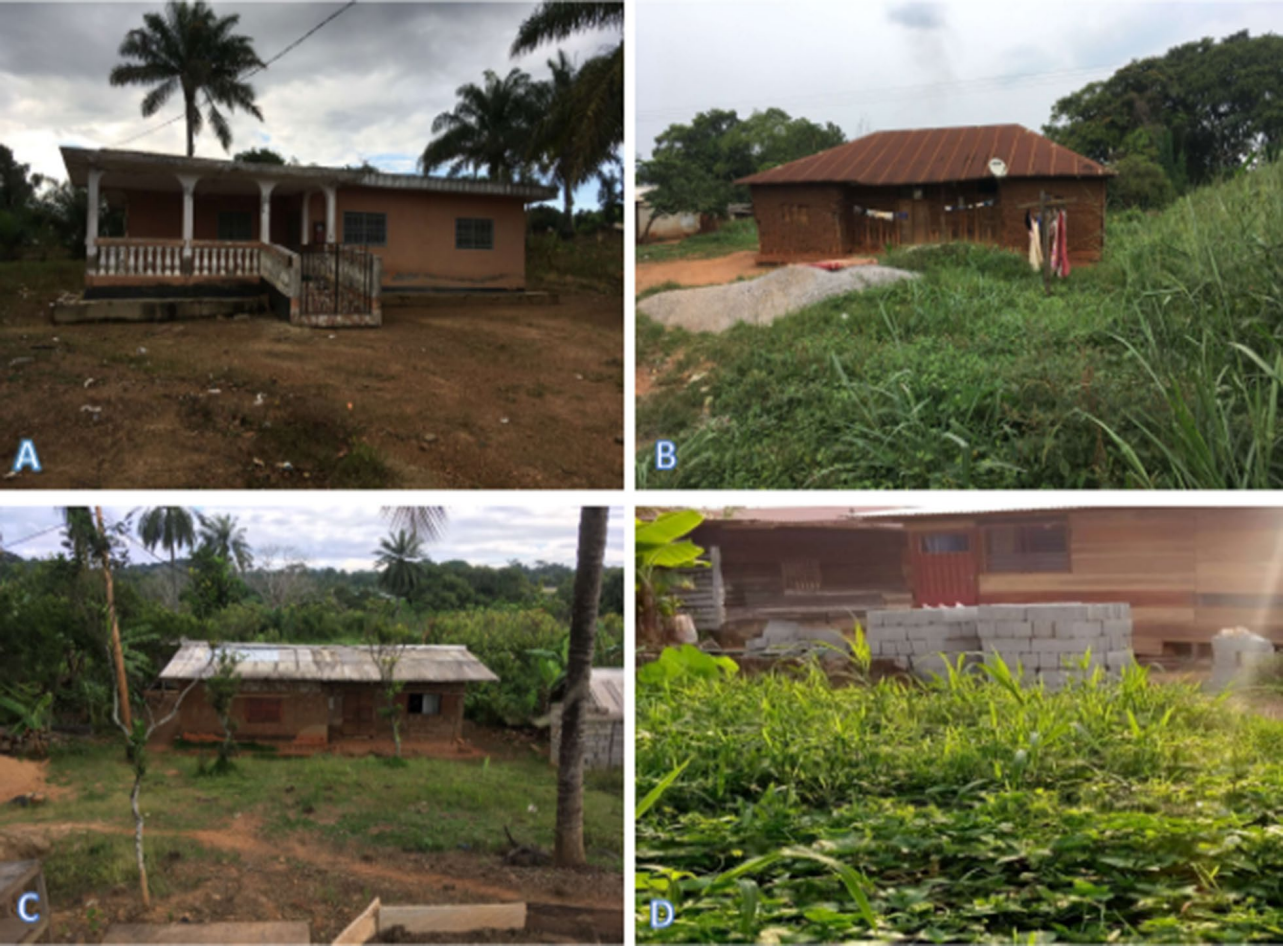
Types of houses in Makenene. Four types were found consisting of **A** cement blocks, **B** Mud and plank, **C** earth brick and **D** plank

A total of 413 households were surveyed and the socio-demographic characteristics of the population are presented in Table 1. The majority of respondents/participants were males (70.94%) of over 40 years old (56.89%). They were predominantly Christians (91.77%) with a secondary school level (68.28%). The heads of households were mostly of small-scale business profession (42.61%) and farmers (33.41%). Different types of houses were found in the locality (Fig. 2) with the majority built in earth brick (76.76%) and without ceiling (68.04%) (Table 1).

**Table 1:** Socio-demographic characteristics of households surveyed in the city of Makenene

Categories	Characteristics	N	%
Gender	Male	293	70.94
	Female	120	29.06
Age (in years)	≤ 40	175	43.10
	≥ 41	231	56.89
Education level	Primary level	64	15.49
	Secondary level	282	68.28
	University level	59	14.29
	None	8	1.93
Religion	Christian	379	91.77
	Muslim	20	4.84
	Other	14	3.39
Occupation of the head of household	Farmer	138	33.41
	Small business	176	42.61
	Civil servant	60	14.53
	Housewife	31	7.51
	Student	8	1.94
Number of people in a household	1 to 5	267	64.65
	6 to 10	130	31.48
	> 10	16	3.87
House construction material	Cement blocks	56	13.56
	Mud and plank	38	9.20
	Earth brick	317	76.76
	Plank	2	0.48
Ceiling	Present	132	31.96
	Absent	281	68.04
Openings between roof and walls	Open	114	27.60
	Close	299	72.4
Grass around house	Present	161	38.98
	Absent	252	61.02
Stagnant water around house	Present	134	32.45
	Absent	279	67.55
Domestic animals	Present	181	43.83
	Absent	232	56.17
Resting places for domestic animals	Inside house	44	22
	Outside house	156	78

General knowledge and behaviour of the study participants about malaria

### General knowledge and behaviour of the study participants about malaria

General knowledge about malaria (the mode of transmission of the disease and preventive measures) of the study participants is shown in Table 2. All respondents declared to have heard about malaria before. The majority of respondents (94.67%) attributed the cause of malaria to mosquito bites and more than 93% complained about mosquito bites in their home. Over 90% of respondent participants (92.25%) stated to use bed nets as a tool of protection against mosquito bites and malaria and most of them declared to use them regularly (77.86%). However, the presence of holes in 68.15% of all mosquito nets independently of the year of acquisition was noted (Table 2). Other protective measures such as sprays and spirals were also used (20.34%).

**Table 2:** Population knowledge and behaviour about malaria in Makenene

Variables	Answers	N	%
Heard about malaria	Yes	413	100
	No	0	0
Mode of transmission of malaria	Mosquito bites	391	94.67
	Dirt	13	3.15
	Other	6	1.45
	No idea	3	0.73
Mosquito bites at home	Yes	387	93.70
	No	26	6.30
Preventive measures^a^	Using mosquito nets	381	92.25
	Using insecticides spray/coil	84	20.34
	Using nets on windows	20	4.84
	Nothing	11	2.66
Period of use of mosquito nets	Rainy season	62	16.15
	Dry season	15	3.91
	Regularly	299	77.86
	Rarely	8	2.08
Origin of bed nets used	Bought	73	18.91
	Freely acquired by MoH^b^	281	72.80
	Donation other than MoH^c^	32	9.29
Reasons for not using mosquito nets regularly	Forgetfulness	62	14.19
	Heat	72	16.47
	Absence of mosquito nets	286	65.45
	Other^d^	17	3.89
Age of bed nets used	< 6 months	36	9.33
	[6 to 12 months]	41	10.62
	[1 to 2 years]	77	19.95
	> 2 years	232	60.10
Presence of holes on bed nets	No	122	31.85
	Yes	261	68.15

*N* number of responses

^a^ Percentages do not add up to 100 because these results are from multiple response questions

^b^ Ministry of Public Health

^c^ Donation by family or hospital

^d^ Other (Insufficient, Muffle, Torn, Crowding)

### Home management of malaria cases by the study participants of Makenene

Out the 413 households surveyed, the majority of participants (51.09%) said not to go to local health centres/hospitals in case of suspected malaria infection. About 30% declared to practice self-medication with conventional drugs bought from roadside sellers, in hospitals or pharmacy while 22.04% used traditional pharmacopoeia. On the other hand, 67.07% of participants said to have suffered from several suspected episodes of malaria from January to August 2021 (Table 3).

**Table 3:** Home management and episodes of malaria cases in households in Makenene from January to August 2021

Items	Characteristics	N	%	p-value
Perceived malaria infections during the year	< 2 times	132	31.96	–
	> 2 times	277	67.07	< 0.001
	No idea	4	0.97	–
Management of malaria cases	Hospital	202	48.91	–
	Traditional	91	22.04	< 0.001
	Self-medication	120	29.05	< 0.001

Ownership and usage of LLINs in households

### Ownership and usage of LLINs in households

A very high LLINs coverage was observed in Makenene (Table 4). In fact, 92.25% of the households surveyed had at least one LLIN, and 51.57% (n = 213) had 1 LLINs for at least 2 people. The LLIN use rate (proportion of people who slept under LLINs the night before the day of the survey) was 86.70%. However, when considering one LLIN per room in households, only 42.85% of houses had all their bed covered.

**Table 4:** LLINs ownership and usage in households in Makenene

Variables	N	%
HHs owning ≥ 1 LLINs	381	92.25
HoHs owning ≥ 1 LLINs for 2 people	2&”	51.57
Persons with access to a LLIN within their own HH	174	42.13
Persons that used LLIN the previous night	358	86.70

*N* number of responses, *HoH* household, *LLIN* long last insecticides treated net

### Factors associated with good knowledge about malaria in Makenene

Knowledge of the disease, the mode of transmission and the symptoms were necessary to assign a participant the qualification of good (56.20%) or bad (43.8%) knowledge. All participants claimed to know malaria and heard about it before. The majority (94.67%) incriminated mosquito bites as the mode of transmission of malaria. Subsequently, univariate and multivariate analyses were carried out in order to determine associations between population good knowledge and some sociodemographic characteristics.

Univariate analyzes showed that Muslims had much better knowledge about malaria than Christians (non adjusted odd ratio [naOR] 3.34; 95% CI: 1.15–11.59; p = 0.03), and participants with a university education level had significantly higher knowledge compared to illiterates (naOR: 7.29; 95% CI 1.37–58.27; p = 0.03) (Table 5). Furthermore, civil servants had better knowledge compared to farmers (naOR: 2.27; 95% CI: 1.12–4.79; p = 0.02). Same results were obtained after multivariate analyses; after adjusting other variables (student, small business, housewife), civil servants still had significantly good knowledge compared to farmers (adjusted odds ratio [aOR] 2.31, 95% CI: 1.14–4.88, p = 0.02). Apart from this, no other significant association between good knowledge and population sociodemographic characteristics was observed after multivariate analyses (Table 5).

**Table 5:** Factors associated with good knowledge about malaria in the study site

Categories	Good knowledge (%)	Bad knowledge (%)	Univariate analysis	Multivariate analysis
			[naOR] (95% CI; p-value)	[aOR] (95% CI p-value)
Gender (Female = Ref)	64 (54.32)	54 (45.77)	–	–
Male	167 (57)	126 (43)	1.08 (0.65–1.79, p = 0.75)	1.07 (0.65–1.7, p = 0.76)
Religion (Christian = Ref)	209 (55.14)	168 (44.32)	–	–
Muslim	15 (75)	5 (25)	3.34 (1.15–11.59, p = 0.03)	4.22 (0.39–47.74, p = 0.21)
Others	5 (35.71)	9 (64.29)	0.99 (0.25–3.86, p = 0.098)	1.23 (0.10–14.99, p = 0.86)
Level of education (Illiterate = Ref)	2 (25)	6 (75)	–	–
Primary level	33 (53.23)	29 (46.77)	3.95 (0.78–30.39, p = 0.12)	0.24 (0.03–1.24, p = 0.11)
Secondary level	154 (54.6)	128 (45.4)	4.42 (0.90–32.25, p = 0.93)	1.04 (0.58–4.88, p = 0.87)
University level	42 (71.19)	17 (28.81)	7.29 (1.37–58.27, p = 0.03)	1.77 (0.79–4.01, p = 0.16)
Occupation (Farmer = Ref)	74 (54.01)	63 (45.99)	–	–
Public servant	46 (76.67)	14 (23.33)	2.27 (1.12–4.79, p = 0.02)	2.31 (1.14–4.88, p = 0.02)
Student	6 (75)	2 (25)	2.45 (0.54–17.25, p = 0.28)	2.43 (0.53–17.13, p = 0.28)
Small business	88 (50.29)	87 (49.71)	0.81 (0.51–1.31, p = 0.52)	0.78 (0.48–1.26, p = 0.32)
Housewife	17 (54.84)	14 (45.16)	1.07 (0.44–2.61, p = 0.88)	1.04 (0.43–2.56, p = 0.91)

[naOR]: Non Adjusted OR; [aOR]: Adjusted OR; OR: Odd ratio; CI: Confidence interval

### Factors associated with good practices about malaria in Makenene

As in the case of population knowledge about malaria, good (71.67%) and bad (28.33%) practices were recorded, and analyses (univariate and multivariate) were carried out with the aim of assessing factors related to good practices of households’ head of Makenene towards malaria prevention and control. No factor was found significantly associated with good practices following univariate analyses except occupation after multivariate analyses (Table 6); public servants had significantly higher good malaria control practices compared to farmers (aOR: 2.31; 95% CI: 1.14 to 4.88; p = 0.02) after adjusting student, small business and housewife.

**Table 6:** Factors associated with practices about malaria in the study sites

Categories	Good practices (%)	Bad practices (%)	Univariate analysis	Multivariate analysis
			[naOR] (95% CI; p-value)	[aOR] (95% CI p-value)
Gender (Female = Ref)	89 (74.17)	31 (25.83)	–	–
Male	207 (70.65)	86 (29.35)	0.80 (0.45–1.39, p = 0.43)	1.07 (0.65–1.7, p = 0.76)
Religion (Christian = Ref)	274 (72.30)	105 (27.70)	–	–
Muslim	14 (0.70)	6 (0.30)	0.75 (0.28–2.25, p = 0.05)	4.22 (0.39–47.74, p = 0.21)
Others	5 (50)	5 (50)	0.38 (0.10–1.47, p = 0.15)	1.23 (0.10–14.99, p = 0.86)
Level of education (Illiterate = Ref)	5 (62.5)	3 (37.5)	–	–
Primary level	45 (70.31)	19 (29.69)	1.54 (0.28–7.27, p = 0.59)	0.24 (0.03–1.24, p = 0.11)
Secondary level	199 (70.57)	83 (29.43)	1.31 (0.25–5.80, p = 0.72)	1.04 (0.58–1.86, p = 0.87)
University level	47 (69.66)	12 (20.34)	2.29 (0.39–11.56, p = 0.32)	1.77 (0.79–4.01, p = 0.16)
Occupation (Farmer = Ref)	91 (65.94)	47 (34.06)	–	–
Public servant	43 (71.67)	17 (28.33)	1.06 (0.52–2.19, p = 0.88)	2.31 (1.14–4.88, p = 0.02)
Student	6 (75)	2 (25)	1.49 (0.32–10.49, p = 0.63)	2.43 (0.53–17.13, p = 0.28)
Small business	136 (77.28)	40 (22.72)	1.68 (1.00–2.84, p = 0.05)	2.43 (0.48–1.26, p = 0.32)
Housewife	20 (64.52)	11 (35.48)	0.75 (0.29–1.95, p = 0.54)	1.04 (0.43–2.56, p = 0.91)

*naOR* non adjusted OR, *aOR* adjusted OR, *OR* odd ratio, *CI* confidence interval

### Factors associated with good attitudes about malaria in Makenene

Analyses were also made to assess any association between good (48.41%) or bad (51.59%) attitudes concerning malaria treatment and participants’ sociodemographic characteristics. Univariate analyses revealed that public servants (naOR: 2.24; 95% CI: 1.12–4.59; p = 0.02) had good attitudes towards malaria compared to farmers and that housewives were less likely to adopt good attitudes towards malaria than farmers (naOR: 0.35; 95% CI: 0.13–0.88; p = 0.03). Same results were obtained with multivariate analyses; after adjusting other variables, public servants (aOR: 2.08; 95% CI: 1.05 to 4.24; p = 0.03) were still more likely to adopt good attitudes in case of malaria infection than farmers, while housewives were less likely to adopt good attitudes than farmers (aOR: 0.33; 95% CI: 0.12 to 0.85; p = 0.02). Apart from occupation (profession), no other factor significantly influenced population good attitudes towards malaria treatment (Table 7).

**Table 7:** Factors associated with good attitudes toward malaria control in the study sites

Categories	Good attitudes (%)	Bad attitudes (%)	Univariate analysis	Multivariate analysis
			[naOR] (95% CI; p-value)	[aOR] (95% CI p-value)
Gender (Female = Ref)	57 (48.31)	61 (51.69)	–	–
Male	141 (48.45)	150 (51.55)	0.77 (0.46–1.27, p = 0.30)	0.76 (0.45–1.26, p = 0.29)
Religion (Christian = Ref)	185 (49.33)	190 (50.67)	–	–
Muslim	10 (50)	10 (50)	1.38 (0.52–3.76, p = 0.51)	4.44 (0.43–104.04, p = 0.24)
Others	2 (20)	8 (80)	0.36 (0.05–1.61, p = 0.22)	1.16 (0.77–32.49, p = 0.91)
Level of education (None = Ref)	1 (12.5)	8 (87.5)	–	–
Primary level	24 (37.5)	40 (62.5)	3.34 (0.63–76.94, p = 0.21)	0.24 (0.01–1.50, p = 0.19)
Secondary level	145 (51.79)	135 (48.21)	6.90 (1.16–131.63, p = 0.07)	1.62 (0.91–2.94, p = 0.10)
University level	28 (49.12)	29 (50.88)	4.59 (0.71–90.82, p = 0.17)	1.16 (0.52–2.58, p = 0.77)
Occupation (Farmer = Ref)	67 (48.55)	71 (51.45)	–	–
Public servant	39 (67.24)	19 (32.76)	2.24 (1.12–4.59, p = 0.02)	2.08 (1.05–4.24, p = 0.03)
Student	7 (87.5)	1 (12.5)	6.62 (1.12–126.07, p = 0.82)	6.51 (1.10–124.06, p = 0.08)
Small business	76 (43.68)	98 (56.32)	0.79 (0.49–1.28, p = 0.33)	0.76 (0.47–1.22, p = 0.27)
Housewife	9 (29.03)	22 (70.97)	0.35 (0.13–0.88, p = 0.03)	0.33 (0.12–0.85, p = 0.02)

*naOR* non adjusted OR, *aOR* adjusted OR, *OR* odd ratio, *CI* confidence interval

## Discussion

The level of knowledge of a population on a given disease is an indicator to guide efficient strategies to its control [35]. In order to understand factors that can hinder people adherence to control tools against malaria and for a better understanding of behaviours likely to promote the transmission of the disease in Makenene, this study assessed knowledge, attitudes and practices of population of Makenene towards the fight against malaria.

Overall, good knowledge, attitudes and practices towards malaria control were observed in Makenene. Almost all heads of households declared to have heard about malaria with over 94% of respondents associating the disease transmission with mosquito bites. This could be linked both to the level of education of the study participants where more than 97% had at least primary level and to the fact that they live in an endemic region. In addition, this endemicity of malaria in the country favors the communication about the disease through different channels such as TV, social media, advertising campaigns, interpersonal discussions and in hospitals through posters/pictograms or discussions with hospital care workers and also sensitization of community health workers [5, 19, 36].

More than half of participants (57.87%) clearly identified at least two symptoms of malaria. Knowledge of symptoms can also be linked to the different sources of information on malaria. Fever appeared to be the most well-known symptom, but it is worth to mention that fever is not necessarily synonymous with malaria. These results are in line with the World Health Organization (WHO) knowledge guidelines [1] and are similar to previous studies [18, 19, 21].

Despite the affordable cost of care in public health hospitals such as in the District Medical Centre of Makenene declare by the government, a high proportion of respondents declared to practice self-medication using commercial drugs and traditional medicines in case of suspected malaria. In fact, there is a schism between the national policy fixing the cost of treatment of malaria and what actually families have to pay in hospitals, which is more higher. Population avoid hospital to limit the cost of treatment from consultations, laboratory testing and drugs. In addition, they believe that going to health service may reduce the time needed for their various activities. This observation was found previously in population living in Yaoundé, Cameroon [5], Tanzania [37] and Bangladesh [38].

With the advance of traditional medicine, people used plants, such as Artemisia (*Artemisia annua*), Quinqueliba (*Combretum micranthum*) and other plants for malaria treatment. Similar results have been found in previous studies [5, 37, 38]. This result could be explained by the fact that, more than half of participants knowing already the main symptoms of the disease (fever, headache, vomiting) have become aware of the routine treatment to take, hence practice self-medication. The over practice of self-medication, the use of traditional drugs coupled with no visit in hospitals may be also due to the fear the population has of visiting health centres in this time of COVID-19 [10, 11].

The lack of knowledge on the doses of traditional drugs, coupled with the unknown quality of drugs from street sellers which are generally stored in poor conditions could be sources of rapid growth in drug resistance [39]. A significant difference was observed when comparing the number of perceived malaria infections made by participants during the period from January to August 2021; about 70% of participants said to have suffered from several episodes of malaria during that period. This could be explained by the fact that malaria is endemic in the locality, patients are in permanent contact with the vector, the environment is appropriate to malaria vectors development, or to a phenomenon of drug resistance. Furthermore, people living in rural settlements usually lack sufficient access to health-care facilities and have in most cases poor housing conditions that expose children to malaria transmitting vectors [40, 41]. In addition, previous studies showed that, the likelihood of malaria episodes increases with the increasing size of the household [42]. The reason for this is that, if there are many individuals in the household as in Makenene where the majority of households had more than four people; when one of them is infected, he/she could serve as reservoir for others [40].

The use of mosquito bed nets was found to play an essential role in preventing malaria infection in African countries [43, 44]. In families where mosquito bed nets were not used when sleeping, were more vulnerable to malaria infection than in families where they were used [41]. In Makenene, more than 92% of respondents said they used it as a means of protection against malaria and 77.86% used it regularly. Similar results have been obtained in studies carried out in some localities in Cameroon [5, 19, 22, 45] and elsewhere [18]. This high possession and regular use of LLINs could be due to the massive distribution campaign of LLINs done by the Ministry of Public Health in 2016 in the locality and sensitization on the usage by community health workers.

Other means of protection, such as insecticide sprays, mosquito coils and screens on windows were also used by the population. Previous studies have shown that the use of insecticide sprays, especially in purely agricultural areas, would increase the rate of resistance in the mosquito population [4, 28, 46] and would, therefore, reduce the bio-efficacy of LLINs [47].

Although LLINs provide both chemical and physical barriers against mosquitoes by reducing their contact with humans, when damaged, they become less effective against mosquito bites. More than 68% of the mosquito nets used in the surveyed households had holes of different sizes. In contrary, results by Talipouo et al. [5] in Yaoundé (Cameroon) reported more than half of the LLINs in use in good condition. Diouf et al. [48] concluded that the rapid degradation of mosquito nets would largely depends on their improper use (washing, drying, threading), which also depends on the year of acquisition. Messages about LLIN washing procedures should be disseminated in the locality to help maintain their integrity, effectiveness and sustainability. This rapid degradation of mosquito nets could also come from the quality of the material used in their manufacture. The materials used in the manufacture of the mosquito nets include cotton, polyethylene, polypropylene, polyester and nylon. Polyester is the most popular because of its lightness and it also allows easy air circulation while, cotton is stronger but less comfortable.

In general, the study showed that more than half of the participants had good knowledge and good practices towards the prevention and control of malaria in Makénéné. Good knowledge and practices were recorded mostly in educated persons including public servants and students. The considerably high rate obtained would therefore constitute an asset for the control of the disease in the locality. Despite this, more than half of the population was found to adopt attitudes not recommended by the WHO when suspecting malaria infection. These results are similar to those obtained by Mbongue et al. [49]. Good attitudes were adopted by public servants and students mostly of secondary and higher levels of education. Similar results were found by others [5, 19, 45]. This testifies the ability of educated people to well practice recommendations received during sensitization campaigns.

The categorization of good knowledge based of only two questions and the use of only one collection tools (questionnaire) are some limitations of this study. In future studies, reasons of answers received from field using in depth interview such as focal group discussion will be seek.

## Conclusion

The level of knowledge and behaviour of populations about a disease is an indicator to guide efficient strategies to fight this disease. The majority of participants in this study claimed to know the mode of transmission of malaria as well as the symptoms of the disease. Good attitudes and practices were also recorded in general with the use of LLINs as the main preventive measure. However, considerable efforts should be made by the public authorities to further raise awareness about care of existing preventive tools and malaria treatment because more than half of the population was found to adopt bad attitudes such self-medication which could contribute to the persistence of the disease in the locality and drug resistance as well.

## Supplementary Information

**Additional file 1.** Questionnaire.

**Additional file 2.** Data base.

## Data Availability

All relevant data are within the paper and its additional information files.

## Abbreviations

LLINs: Long-lasting insecticidal nets
KAP: Knowledge, Attitude and Practice
NMCP: National Malaria Control Programme
COVID-19: Corona virus 2019
WHO: World Health Organization
CHWs: Community health workers
HoH: Household
