# Supplementary material for: Population knowledge, attitudes and practices towards malaria prevention in the locality of Makenene, Centre-Cameroon
Source: Malar J. 2022 Aug 5;21:234. doi: 10.1186/s12936-022-04253-z (PMC9356395; doi:10.1186/s12936-022-04253-z)
Supplement: Supplementary file 1 — Additional file 1. Questionnaire. [file 12936_2022_4253_MOESM1_ESM.pdf]

FICHE D’ENQUETE MENAGE SUR LE PALUDISME A MAKENENE

Investigateurs : - Dr Bamou Roland (Université de Dschang ; tel : 655101080)

Djoufounna Joël (Université de Dschang ; tel : 657383259)

But : Identifier les paramètres comportementaux modulant le niveau de transmission du paludisme

Les informations collectées au cours de cette enquête sont strictement confidentielles au terme de la Loi N°91/023 du 16 Décembre 1991 sur les Recensements et Enquêtes Statistiques qui stipulent en son article 5 que « les renseignements individuels, d’ordre économique, et/ ou financier figurant sur tout questionnaire d’enquête statistique ne peuvent en aucun cas être utilisés à des fins de contrôle ou de répression économique »

IDENTIFICATION

**0.1**	Date	|___||___|/|___||___|/2021	
**0.2**	Enquêteur/numéro du questionnaire		|___||___|
**0.3**	Localité/Quartier		|___||___|
**0.4**	N° Maison		|___||___|
**0.5**	Coordonnées GPS	**A.** Latitude<br>**B.** Longitude	|___||___||___||___||___||___| |___||___||___||___||___||___|
**0.6**	Sexe du chef de ménage/Age	**A.** Masculin |___| **B.** Féminin |___|	**Age** |_______|
**0.7**	Profession du chef de ménage/famille	____	
**0.8**	Statut matrimoniale	**A.** Marié |____| **B**. Célibataire |____|	
**0.9**	Niveau d’étude du chef de ménage/famille	**A.** Université |____|; **B.** Secondaire|____| ;<br>**C.** Primaire |____|; **D.** Sans niveau|____|	
**0.10**	Religion du chef de ménage	**A.** Musulman |___| ; **B**. Chrétien |___|;<br>**C.** Autres |_____________|	
**0.11**	Le répondant est-il le chef du ménage?	**A.** Oui |___| **B.** Non |___|	
**0.12**	Niveau d’étude du répondant	**A.** Université |____|; **B.** Secondaire |___|;<br>**B.** Primaire |____|; **D.** Sans niveau|___|	
**0.13**	Sexe du répondant	**A.** Masculin |____| **B**. Féminin |____|	
**0.14**	Quel est le nombre de personnes vivant dans la maison ?	**A.** Effectif total |_______| **B.** Enfants de moins de 5 ans |_______|	

CONNAISSANCES, ATTITUDES, PRATIQUES VIS-À-VIS DU PALUDISME

**1.1**	Connaissez-vous le paludisme ? Oui |__| ;<br>Non |__|. Si oui , où avez-vous entendu parler ?	**A .** Radio |___| **;** **B.** Télévision |____| ;<br>**C.** Réseaux sociaux |___| **D.** Ecole |___| ;<br>**E.** Autres |___________________________________|

**1.2**	D'après vous qu'est ce qui transmet le paludisme à l'homme ?	____ ___
**1.3**	Les moustiques vous piquent-ils dans votre maison ?	**A.** Oui |____| ; **B**. Non |____|
**1.4**	A quel moment vous piquent-ils ? Préciser l'heure	**A..** Matin |__________| ; **B.** En journée |______| ;<br>**C.** En soirée |_________| ; **D.** La nuit |________|
**1.5.**	Que faites-vous en général à ce moment ?	____
**1.6**	Quelle(s) est (sont) alors la (les) mesure(s) de protection que vous utilisez contre les piqûres des moustiques ?	**A.** Moustiquaires |____| **B.** Grilles anti moustiques aux fenêtres |___| **C.** Repellents |____| **D.** Ventilateur |____| **E.** Spirales |____| **F.** Pulvérisation d'insecticides |____| **G.** Climatiseur |____| Autre (à préciser) __________________
**1.7**	Si vous utilisez une moustiquaire, à quelle période l'utilisez-vous plus ?	**A** .Saison des pluies |___| ; **B.** Saison sèche |___| ;<br>**C** . Régulièrement |___| ;<br>**D** .Autre (à préciser) __________________________________
**1.8**	Depuis combien de temps avez-vous votre (vos) moustiquaire (s) ?	**A** .Moins de 6 mois |___| ; **B**. Plus de 6 mois |___| ;<br>**C** . Plus d'un an |___| ; **D**. Plus de 2 ans |___| ;
**1.9**	Comment avez-vous obtenu votre (vos) moustiquaire (s) ?	**A .** Distribution du MINSANTE |___| ;<br>**B .** Achat |___| ;<br>**C .** Don (autre que le MINSANTE) |___| ;<br>**D** .Autre à préciser______________________________
**1.10**	Marque de votre moustiquaire ?	___________________________
**1.11**	Tous les lits à la maison ont-ils des moustiquaires imprégnées ?	**A** .Oui |___| **B.** Non |___|
**1.12**	Combien de moustiquaires avez-vous ?	_____________________________________
**1.13**	Combien de chambres compte votre maison ?	________________________________
**1.14**	Combien de lits avez-vous ? (unité de couchage)	___________________________________

**1.15**	Quels sont ceux qui dorment régulièrement sous une moustiquaire à la maison ?	**A.** Tout le monde |___| **B.** Parents |___| **C.** Tous les enfants |___| **D.** Enfant de <5ans |___| **E.** Visiteurs |___| **F.** Autre (à préciser_____________________________________
**1.16**	Parmi les réponses de la question précédente, qui a dormi sous moustiquaire la nuit dernière ?	**A.** Tout le monde |___| **B.** Parents |___| **C.** Tous les enfants |___| **D.** Enfant de <5ans |___| **E.** Visiteurs |___| **F** .Autre (à préciser) ___________________________________
**1.17**	Ceux qui n'utilisent pas la moustiquaire ne le font pas pourquoi ?	**A .** Oubli |____| ; **B.** Chaleur |____| ;<br>**C** . Pas de moustiques à la maison |____| ;<br>**D** .Autre (à préciser) _____________________________________
**1.14**	Niveau de délabrement de votre (vos) moustiquaire(s) ?	**A** . En bon état (sans trous) |____| ;<br>**B** .Endommagée(s) (présence des trous) |____| ;
**1.15**	Pensez-vous réellement que les moustiquaires protègent contre le paludisme ?	**A** .Oui |___| ;<br>**B** .Non |___| :pourquoi ?_________________________________
**1.16**	Selon vous quels sont les symptômes du paludisme ?	Citer :
**1.17**	Que faites-vous quand vous suspectez un cas de paludisme ?	**A .**Hôpital pour consultation |____| ;<br>**B .** Automédication |____| ;<br>**C .** Pharmacopée traditionnelle |____|
**1.18**	Combien de fois avez-vous souffert du paludisme cette année ?	**A** .Moins de 2 fois |____| ; **B.** Plus de 2 fois |____|

ACTIVITES NOCTURNES

**2.1**	Les activités nocturnes de la famille (Lister les activités)<br>***Nb : à partir de 19h***	Femmes____<br>_________________<br>Hommes____<br>___________________<br>Garçons____<br>____<br>___________________<br>Filles____<br>__________________

**2.2**	Quelles sont les heures de coucher et de réveil ?	Père [ ] [ ]<br>Mère [ ] [ ]<br>Enfants (0-5 ans) [ ] [ ]<br>Enfants (6-14 ans) [ ] [ ]<br>Enfants (>15 ans) [ ] [ ]

HABITATS ET ENVIRONS

**3.1**	Type habitat (observation)	**A** .Dur |___| ; **B** .Semi dur |___| ;<br>**C** .Planche |___| ; **D** .Terre battue |___| ;<br>**E .** Autres (Spécifier)________________________________
**3.2**	Type de toît	**A** .Aluminum |___| ; **B .** Paille |___| **C** . Autres____
**3.3**	Présence d'avant- toit	**A .** Ouvert |____| ; **B .** Fermé |____|
**3.4**	Présence du plafond	**A** .Oui |____| ; **B** .Non |____|
**3.5**	Y a-t-il des animaux dans la maison ?	**A** .Oui |____| ; **B .** Non |____|
**3.6**	Où sont gardés les animaux pendant la nuit ?	**A** .Dans des magasins à l'intérieur des maisons |___| **B.** Dans les mêmes pièces que la famille |___| **C**. Hors de la maison /enclos |___| **D** .Autres ____
**3.7**	En quoi sont fait les murs de votre maison ?	**A.** Terre et bois |___| **B.** Boue/planche/pierre |___| **C.** Boue / pierre |___| **D.** Planche |___| **E.** Pierre |___| **F.** Brique |___| **G.** Autre ___________________________________
**3.4.**	Y'a-t-il des ouvertures entre le toit et les murs?	**A.** Oui |____| **B**. Non |____|
**3.5**	Y' a-t-il des eaux stagnantes aux alentours de votre maison ?	**A.** Oui |____| **B.** Non |____|
**3.6.**	Présence/Absence de la broussaille aux alentours de la maison	**A.** Présence |____| **B.** Absence |____|

Souhaitez-vous qu'on remplace votre moustiquaire ? |______| Si oui tel : |___||___||___||___||___||___||___||___|

Motif du remplacement |____|
